# CD28 Co-Stimulation Down Regulates Th17 Development

**DOI:** 10.1371/journal.pone.0005087

**Published:** 2009-03-31

**Authors:** Salim Bouguermouh, Geneviève Fortin, Nobuyasu Baba, Manuel Rubio, Marika Sarfati

**Affiliations:** Immunoregulation laboratory, CHUM Research Center, Notre-Dame Hospital, Montreal, Quebec, Canada; New York University School of Medicine, United States of America

## Abstract

Th17 cells are implicated in host defence and autoimmune diseases. CD28/B7 co-stimulation is involved in the induction and progression of autoimmune diseases, but its role in controlling murine Th17 cell fate remains to be clarified. We here report that soluble anti-CD28 mAb suppressed the differentiation of anti-CD3-stimulated naïve CD4^+^ T cells into IL-17-producing cells. CD28 co-stimulation reduced the frequency of proliferating cells that produce IL-17. We provide evidence for an IL-2 and IFN-γ-dependent mechanism of CD28-mediated IL-17 suppression. CD28 blockade of Th17 development was correlated with a decrease rather than an increase in the percentage of Foxp3^+^ T cells. In APC/T cell co-cultures, mature dendritic cells (DC) were less efficient than immature DC in their ability to support Th17 cell differentiation, while CTLA4-Ig, an agent blocking CD28/B7 and CTLA4/B7 interactions, facilitated both murine and human Th17 differentiation. This study identifies the importance of B7 co-stimulatory molecules in the negative regulation of Th17 development. These unexpected results caution targeting the CD28/B7 pathways in the treatment of human autoimmune diseases.

## Introduction

Th17 cells play a central role in autoimmune inflammatory conditions such as multiple sclerosis (MS) [1], rheumatoid arthritis (RA) [2] and Crohn's disease [3]. Th17 cells are also involved in the protection against several pathogens including *Klebseilla pneumoniae, Staphylococcus aureus, Mycobacteria tuberculosis* and *Candida albicans* [4]. These cells are characterised by the expression of the transcription factor RORγt and the production of the cytokines IL-17, IL-22, IL-6 and tumor necrosis factor-α (TNF-α). The *in vitro* differentiation of the murine Th17 cell lineage depends on the synergy between transforming growth factor-β (TGF-β) and IL-6, combined with the neutralization of IFN-γ and IL-4. TNF-α and IL-1β act as amplifiers, while IL-23 stabilizes the Th17 phenotype [5], [6], [7], [8], [9]. The combination of TGF-β and IL-21 constitutes an alternative pathway for Th17 development [10]. The activation and differentiation of CD4^+^ T helper lymphocytes into Th1 or Th2 cells requires both T cell receptor/MHC-peptide specific recognition and co-stimulatory signals. The best-defined co-stimulatory pathway involves the B7 family, where B7.1 (CD80) and B7.2 (CD86) molecules on antigen presenting cells interact with CD28 and CD152 (CTLA4) on T cells [11], [12], [13]. However, the precise role of these co-stimulatory molecules in the regulation of Th17 development remains unclear. The rationale for assessing the role of CD28 signalling in Th17 differentiation stemmed from reports demonstrating that treatment of experimental autoimmune encephalomyelitis (EAE) with anti-CD80 or anti-CD86 Abs or multiple injections of CTLA4-Ig resulted in enhanced disease severity [14], [15]. Additionally, B7 deficiency facilitates autoimmunity, and this, among other possible mechanisms, was attributed to a loss or inactivation of regulatory T cells [12], [16]. Moreover, the development of diabetes in Non-obese Diabetic (NOD) mice is exacerbated by deletion of CD28 [17].

Finally, the clinical report of the development of ulcerative colitis during CTLA4-Ig (abatacept) therapy in a patient with RA reinforces our hypothesis that costimulatory molecules may regulate human Th17 development [18]. We here demonstrate that CD28 signalling suppresses Th17 differentiation, while CTLA4-Ig blockade promotes mouse and human Th17 polarization *in vitro*.

## Materials and Methods

### Mice

All 8- to 12-week-old BALB/c mice were housed in our breeding colony and animal care facility under specific pathogen-free conditions. All experimental protocols were approved by the Centre de Recherche du Centre Hospitalier Universitaire de Montréal (CRCHUM) and the Canadian Council on Animal Care.

### Culture medium, Antibodies and Reagents

Mouse cells were cultured in complete RPMI-1640 medium (Wisent Inc., St-Bruno, QC, Canada) supplemented with 10% fetal bovine serum (FBS), Penicillin (500 U/ml), Streptomycin (500 µg/ml), HEPES buffer (10 mM), and 2-ME (1 mM), (GIBCO-BRL, Invitrogen corp., Carlsbad, CA, USA). LPS (*E.Coli*) was obtained from Sigma-Aldrich (St. Louis, MO, USA). Recombinant mouse IL-1, IL-6 and recombinant human TGF-β1 were purchased from R&D Systems (Minneapolis, MN, USA). Recombinant mouse GM-CSF and anti-murine IL-2 were purchased from PeproTech (Rocky Hill, NJ, USA). Mitomycin-C was purchased from EMD Chemicals (Gibbstown, NJ, USA). The following clones were purchased from American Type Culture Collection (Rockville, MD, USA) and purified in our laboratory: GK1.5 (anti-CD4, biotinylated); R46A2 (anti-IFN-γ); 11B11 (anti-IL-4), IM7.8.1 (anti-CD44, biotinylated). Allophycocyanin, FITC or PerCP-conjugated anti-mouse CD4, PerCP-conjugated streptavidin, PE-conjugated anti-mouse CD40, CD86 and IA/IE, allophycocyanin-conjugated Annexin V, allophycocyanin-conjugated anti-mouse IL-2 and IFN-γ, anti-mouse CD28 and purified mouse CTLA4-Ig fusion protein were purchased from BD Biosciences (Mississauga, ON, CA). PE- or allophycocyanin-conjugated anti-mouse IL-17A, APC/Cy7-conjugated anti-mouse CD25, APC/Cy7 or PE/Cy7-conjugated streptavidin and anti-mouse CD3 were purchased from Biolegend (San Diego, CA, USA). Recombinant murine IL-23 and anti-mouse Foxp3-PE staining kits were purchased from eBioscience (San Diego, CA, USA). PE-conjugated anti-mouse CD62L was purchassed from Caltag Laboratories (Burlingame, CA, USA). Carboxyfluorescein diacetate succinimidyl ester (CFSE) was purchased from Invitrogen (Carlsbad, CA, USA).

### Cell purification and *in vitro* generation of Th17 cells

Total CD4^+^ T cells were purified from spleen and peripheral lymph nodes (LNs) by positive selection using immunomagnetic beads according to the manufacturer's protocol (EasySep Biotin Selection Kit, StemCell Technologies Inc, Vancouver, BC, CA). For some experiments, naïve CD4^+^ (CD4^+^CD62L^high^CD44^low^), naïve CD4^+^CD25^−^ (CD4^+^ CD62L^high^CD44^low^CD25^−^) and memory CD4^+^ (CD4^+^CD62^low^CD44^high^) T cells were isolated from splenocytes and peripheral lymph nodes (LNs) by sorting using a Becton Dickinson FACSAria II cell sorter (BD Bioscience). Cells (1×10^6^ cells/ml) were activated with plate-bound anti-mouse CD3 (5 or 10 µg/ml) in flat bottom 96-well plates in the presence of the Th17-promoting cytokine cocktail IL-1-α, IL-6, IL-23 (10 ng/ml) and TGF-β1, (1 ng/ml) with or without anti-mouse CD28 mAb (2 µg/ml). For some experiments, 10 µg/ml of neutralizing anti-mouse antibodies (anti-IL-2, anti-IFN-γ and anti-IL-4) were added to the cultures. After 4–5 days, recovery of viable cells was assessed by trypan blue (GIBCO-BRL, Invitrogen corp.) exclusion. The cells were restimulated during 6 h with PMA/ionomycin. For Treg cell experiments, total CD4^+^ T cells, CD4^+^CD25^−^ and CD4^+^CD25^+^ T cells were separated from spleen or peripheral LNs using the EasySep Negative Selection Mouse CD4^+^ T cell Enrichment Kit followed by the mouse CD25-Microbeads Kit (Miltenyi Biotec, Auburn, CA, USA) according to the manufacturer's protocols. CD4^+^CD25^−^ T cells (1×10^6^ cells/ml) were cultured alone or in the presence of CD4^+^CD25^+^ T cells at two different CD4^+^CD25^−^/CD25^+^ ratios (1/1, 4/1) for 4–5 days and activated on anti-CD3 (10 µg/ml) coated plates under Th17 conditions in the presence or the absence of anti-mouse CD28 mAb.

### Culture of CD4^+^ T cells with BMDC

Bone marrow-derived DC (BMDC) were generated as described previously [19]. In some experiments, BMDC were activated overnight with 1 µg/ml of LPS (mBMDC) and stained for CD86, CD40, or MHCII (IA/IE) expression. CD4^+^ T cells (2.5×10^5^ cells/ml) were purified from spleens as described and stimulated with soluble (2 µg/ml) or coated anti-CD3 (5 µg/ml) in the presence of immature (untreated) or mature (treated with LPS) mitomycin-C-treated BMDC at different CD4^+^/BMDC ratios (2/1, 1/1, 1/2). The cultures were supplemented with the Th17 promoting cytokine cocktail. In some experiments, mouse CTLA4-Ig (20 µg/ml) fusion protein was added to CD4^+^/mBMDC cultures.

### Intracellular cytokine staining

For intracellular staining, cells were restimulated for 6 h with PMA (phorbol 12-myristate 13-acetate (PMA, 10 ng/ml) and ionomycin (1 µg/ml) in the presence of brefeldin A (1 µg/ml) per 1×10^6^ cells. Cells were stained in the presence of Fcγ Blocker (Clone 24G2) with FITC, PerCP or allophycocyanin-conjugated anti-CD4 antibodies, fixed and permeabilized (BD Cytofix/Cytoperm, BD Biosciences or eBioscience for Foxp3 staining). Cells were assessed for IL-17, IFN-γ, IL-2 and Foxp3 expression. Data were acquired on a Becton Dickinson FACS Calibur and analyzed with Cell Quest software (BD Bioscience).

### Labeling Naïve CD4^+^CD25^−^ T cells with CFSE

Naïve CD4^+^CD25^−^ (CD4^+^CD62L^high^CD44^low^CD25^−^) T cells were isolated from splenocytes and peripheral lymph nodes (LNs) by sorting using a Becton Dickinson FACSAria II cell sorter (BD Bioscience). CD4^+^ T cells were washed and resuspended at final concentration of 10^7^/mL in EasySep buffer (PBS containing 2% FBS and 1 mM EDTA). CFSE was added at a final concentration of 1 µM and incubated for 15 min at 37 degree. The reaction was stopped by washing cells twice with RPMI 1640, containing 10% FBS. CFSE-labeled naïve CD4^+^CD25^−^ T Cells (1×10^6^ cells/ml) were activated as previously described with plate-bound anti-mouse CD3 under Th17 conditions with or without anti-mouse CD28 mAb. The cells were monitored on day 4–5 for CFSE cell dilution and cytokine expression.

### Assessment of apoptosis

The CD4^+^ cells were stimulated as described with anti-CD3 in the absence or presence of anti-CD28, harvested from cultures at 48 h, 72, 96 and 120 h, to monitor apoptosis. Briefly, cells were washed and resuspended in 100 µl of Annexin binding buffer (10 mM HEPES, 150 mM NaCl, 5 mM KCl, 1 mM MgCl_2_, 1.8 mM CaCl_2_) at 1×10^6^/ml with 5 µl of allophycocyanin-conjugated Annexin V (BD Biosciences) and incubated 5 minutes at room temperature. The samples were then analyzed by flow cytometry.

### ELISA

The production of IL-17 and IFN-γ was measured with the IL-17 Duoset ELISA kit (R&D Systems) and the OptEIA Mouse IFN-γ ELISA set (BD Biosciences), respectively, according to the manufacturer's protocols.

### Quantitative RT-PCR analysis

Total RNA was extracted from cell cultures using the RNeasy mini kit (Qiagen, Mississauga, ON) and reverse-transcribed using the cDNA reverse transcription kit (Applied Biosystems, Foster City, CA). Quantitative real-time PCR was performed using an ABI Prism 7900HT Sequence Detection System (Applied Biosystems) (1 PCR cycle, 95°C, 10 minutes; 40 PCR cycles, 60°C, 1 minute, 95°C, 15 seconds). cDNA was amplified in a 10 µl final reaction mix containing TaqMan Universal PCR Master Mix (Applied Biosystems) and corresponding TaqMan® Gene Expression Assays (Mm00444241_m1 (IL-22), Mm00439619_m1 (IL-17a), Hs99999901_s1 (Eukaryotic 18s rRNA), Applied Biosystems). Signals were analyzed by the ABI Prism Sequence Detection System software version 2.2 (Applied Biosystems). The comparative Ct method for relative quantification was used, whereby all threshold cycles (Ct) are first normalized to the expression of an endogenous control (18s rRNA). The normalized values were then divided by the average delta Ct value of anti-CD3-stimulated samples. Here, the cytokine expression is represented as a fold-change relative to CD3-stimulated cells±SEM.

### Human DC/T co-cultures

Human PBMC were obtained from healthy volunteers in compliance with the Institutional (CR-CHUM) Research Ethics Committee. Naïve CD4^+^ T were isolated from total PBMC using the human CD4^+^ Naïve T cell Enrichment Kit (Stem Cell Technologies). The purity of adult human naïve CD4^+^ T cells was >99% CD4^+^CD45RA^+^ and comprised <0.5% single CD45RORA^+^ cells as assessed by flow cytometry analysis. Naïve CD4^+^ T cells were co-cultured for 5 days with mature monocyte-derived DC at a DC/T ratio of 1/25 in Yssel's medium (Gemini Bio-product, West-Sacramento, CA, USA) supplemented with 2% human AB serum (Wisent) in the presence of 100 ng/ml of soluble anti-CD3 (OKT-3; Janssen-Ortho) with or without human CTLA4-Ig fusion protein (5 µg/ml) (Abatacept, Orencia, Bristol-Myers Squibb, Canada) for 5 days. Primed T cells were expanded for 5 days in the presence of IL-2 (20 U/ml, R&D Systems). T cells were restimulated for 6 h with PMA (5 ng/ml) and ionomycin (0.5 µg/ml) in the presence of monensin (3 µM). Intracytoplasmic staining was performed using BD Cytofix/Cytoperm and anti-IL-17-APC (R&D System). Culture supernatants were collected after re-stimulation and IL-17, IL-22 and IFN-γ were detected by ELISA (R&D Systems).

### Statistical Analysis

Statistical analyses were performed using the GraphPad Instat program.

## Results

### CD28 co-stimulation negatively regulates the development of naïve CD4^+^ (CD4^+^CD62L^high^CD44^low^) T cells into IL-17-producing T helper cells

**Figure 1:**
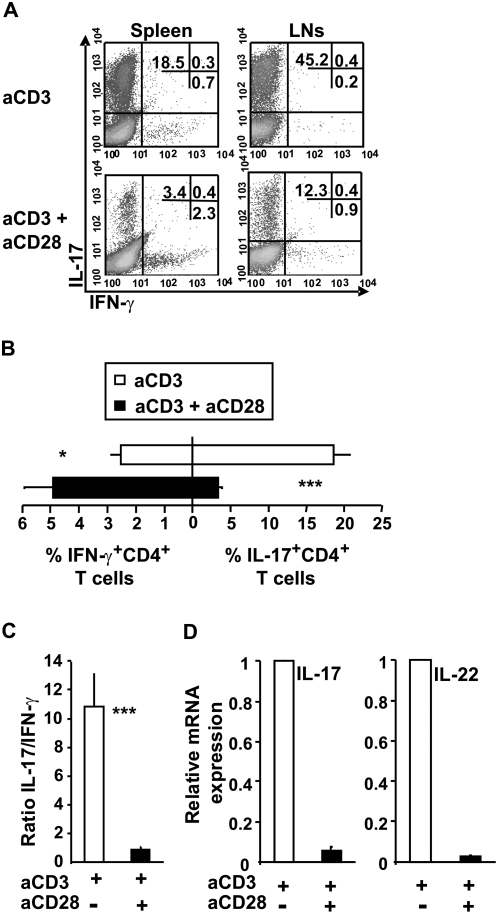
CD28 co-stimulation inhibits *in vitro* generation of IL-17 producing CD4^+^ T cells. Purified CD4^+^ T (1×10^6^) cells from spleen (A–C) and LNs (A) were stimulated with plate-bound anti-CD3 with or without anti-CD28 under pro-Th17 conditions for 4–5 days and restimulated for 6 h with PMA/Ionomycin in the presence of brefeldin A. (A) CD4^+^ T cells were stained intracellularly for IL-17 and IFN-γ. Numbers represent the percentage of events in each quadrant. (B) Cumulative data of the percentage of CD4^+^IL-17 and IFN-γ positive T cells after restimulation as assessed by intracytoplasmic staining. Data represent the mean±SEM of at least ten independent experiments. (C) The ratio of the percentage of IL-17^+^ to IFN-γ^+^ cells among CD4^+^ T cells. (D) qRT-PCR analysis of IL-17 and IL-22 mRNA expression in purified CD4^+^ T cells cultured as described in A. Data are expressed as mean±SEM of four experiments. P values were calculated using the two-tailed, paired Student's *t* test, *, P<0.05; ***, P<0.001.

**Figure 2:**
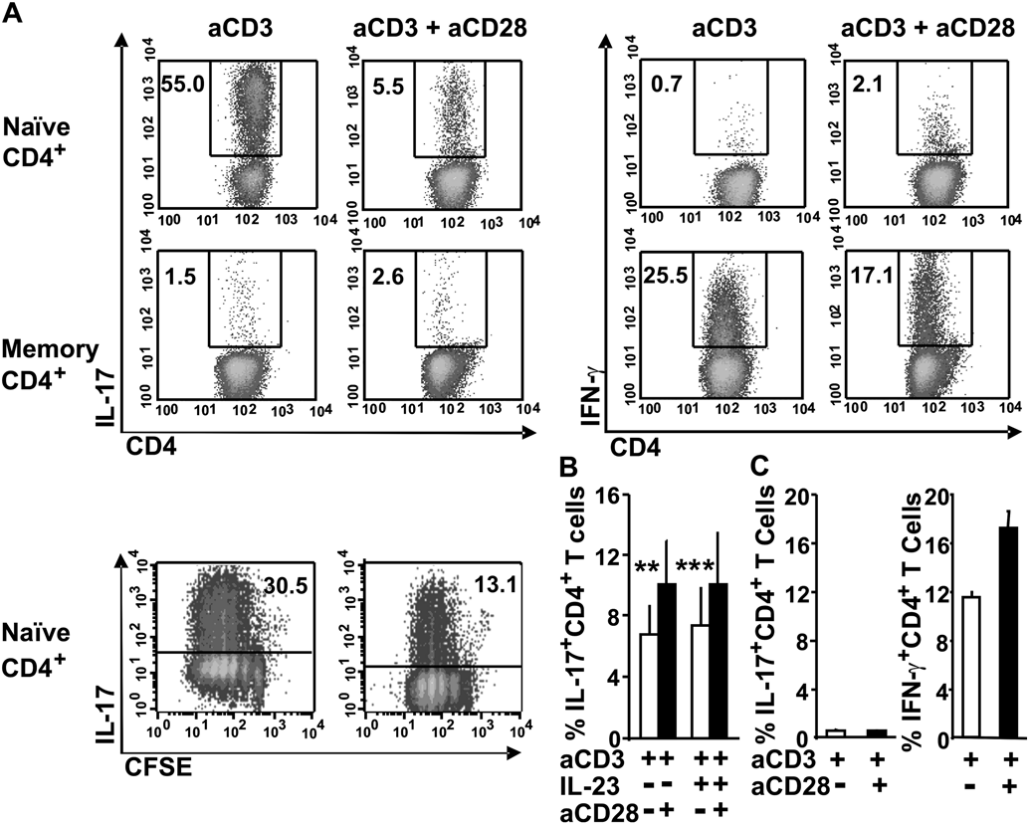
CD28 co-stimulation did not modulate memory Th17 cells. (A) FACS sorted naïve CD4^+^ (CD4^+^CD62L^high^CD44^low^), CFSE-labeled naïve CD4^+^ CD25^−^ (CD4^+^CD62L^high^CD44^low^CD25^−^) and memory CD4^+^ (CD4^+^CD62L^low^CD44^high^) T cells were cultured with anti-CD3 in the presence or absence of anti-CD28 mAb under pro-Th17 conditions for 4–5 days. Shown is one representative experiment out of at least three. (B) CD4^+^ T cells were stimulated with anti-CD3 under Th17 polarizing conditions for 4–5 days and restimulated with plate-bound anti-CD3 in the presence or absence of IL-23, and with or without anti-CD28 mAb for 2 days. Data represent the mean±SD of five to seven independent experiments. (C) Purified CD4^+^ T cells were cultured with anti-CD3 in the presence or absence of anti-CD28 mAb without adding Th17 differentiation cytokines for 4–5 days. Data are the mean±SD of 2 independent experiments. (A–C) Cells were restimulated for 6 h with PMA/Ionomycin in the presence of brefeldin A. Naïve and memory CD4^+^ T cells were stained intracellularly for IL-17 and IFN-γ and CFSE-labeled naïve CD4^+^CD25^−^ for Il-17. Numbers represent the percentage of positive cells in each gate. P value were calculated using the two-tailed, paired Student's *t* test, **, P<0.01; ***, P<0.001.

We here hypothesised that the CD28/B7 co-stimulatory pathway controls Th17 development. We first examined the effect of anti-CD28 mAb on Th17 differentiation and cytokine mRNA expression. CD4^+^ T cells isolated from the spleen or peripheral LNs of wild-type BALB/c mice were activated on plate-bound anti-CD3 under Th17 polarizing conditions in the presence or absence of soluble anti-CD28 mAb. After 4–5 days of primary culture, cells were re-stimulated with phorbol 12-myristate 13-acetate (PMA) and ionomycin. As depicted in Fig. 1, anti-CD28 mAb strongly inhibited IL-17 production by anti-CD3 stimulated CD4^+^ T cells as determined by intracellular cytokine staining and enzyme-linked immunosorbent assay (ELISA) (Fig. 1, A–B and data not shown). Accordingly, anti-CD28 mAb diminished the proportion of IL-17-producing cells by 82.8% (mean inhibition±8.1%, n = 14). In contrast, IFN-γ production was significantly increased as reflected by a decreased IL-17/IFN-γ ratio after CD28 engagement (Fig. 1C). Quantitative real-time PCR (qRT-PCR) analysis of mRNA revealed a reduction in IL-17 and IL-22 expression (Fig. 1D). Since CD4^+^ T cells are comprised of ∼90% naïve (CD4^+^CD62L^high^CD44^low^) and ∼10% of memory (CD4^+^CD62L^low^) T cells, we next compared naïve and memory T cells for their ability to differentiate into Th17 in the presence or absence of CD28 mAb. We showed that CD28 engagement strongly inhibited naïve T cell differentiation into Th17 without preventing their proliferation (Fig. 2A). Indeed, IL-17 was exclusively produced by dividing cells and CD28 co-stimulation decreased the proportion of proliferating cells that produced IL-17. In contrast, *ex vivo* isolated memory T cells represented a minor source of IL-17 (less than 2% IL-17^+^CD4^+^ T cells) that was not modulated by CD28 mAb (Fig. 2A). Under Th17 polarizing conditions, memory T cells produced large amounts of IFN-γ when compared to naïve T cells.

We next verified whether CD28 engagement further regulates T cells that have been differentiated into Th17 with anti-CD3 in the absence of co-stimulation. To this end, CD4^+^ T cells activated during 4–5 days with coated anti-CD3 under Th17 conditions were restimulated with anti-CD3 and IL-23 in the presence or absence of anti-CD28 mAb. Interestingly, we found that anti-CD28 mAb significantly enhanced IL-17 production upon re-stimulation, demonstrating that CD28 co-stimulation did not inhibit fully differentiated Th17 cells (Fig. 2B). As expected, anti-CD3-activated CD4^+^ T cells were not polarized into Th17 cells in the absence of the pro-Th17 cocktail, but instead resulted in the development of IFN-γ−producing T cells, which was further augmented by CD28 co-stimulation (Fig. 2C). We conclude that anti-CD28 mAb inhibits naïve CD4^+^ T cells polarization into Th17.

### TCR avidity and CD28 co-stimulation regulates Th17 development

**Figure 3:**
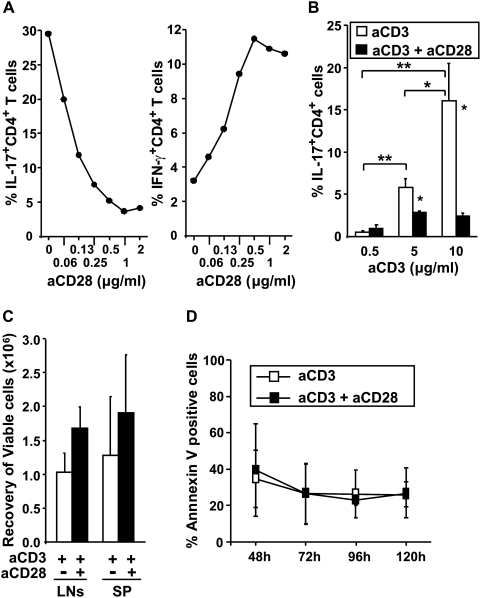
TCR avidity and CD28 co-stimulation signals regulate Th17 development. (A) Purified CD4^+^ T were stimulated with plate-bound anti-CD3 (5 µg/ml) and titrated doses of soluble anti-CD28 mAb under Th17 polarizing conditions. Cells were stained intracellularly for IL-17 and IFN-γ expression. The data shown is one representative experiment out of two. (B) CD4^+^ T cells were stimulated with titrated doses of plate-bound anti-CD3 in the presence or absence of soluble anti-CD28 mAb (2 µg/ml). Data represent the mean±SD of four independent experiments. (C) Cumulative data of viable CD4^+^ T cell recovery after stimulation with anti-CD3 (5 µg/ml) the presence or absence of anti-CD28 mAb (2 µg/ml). Data are the mean±SD of 3 (LNs) to 20 (spleen) independent experiments. (D) CD4^+^ T cells were stimulated with coated anti-CD3 (10 µg/ml) in the presence or absence of soluble anti-CD28 under Th17 conditions and analyzed at different time points for the percentage of apoptotic cells (Annexin V binding positivity). Mean values±SD obtained from at least three separate experiments are presented. P values were calculated using the two-tailed, paired Student's *t* test, *, P<0.05; **, P<0.01.

We next determined the EC_50_ of anti-CD28 mAb (effective concentration of anti-CD28 mAb resulting in a 50% inhibition of IL-17 secretion) on Th17 differentiation by supplementing CD3-stimulated T cells with increasing doses of mAb. Suppression was observed with concentrations as low as 0.06 µg/ml, Th17 development was almost entirely abrogated at 1 µg/ml and the EC_50_ of anti-CD28 mAb was 0.1 µg/ml (Fig. 3A, left). The enhancement in IFN-γ secreting CD4^+^ T cells was inversely correlated with the frequency of IL-17-producing cells (Fig. 3A, right). To examine whether impairment of IL-17 production with anti-CD28 mAb was dependent on the TCR-signalling intensity, CD4^+^ T cells were stimulated with titrated doses of anti-CD3 and a fixed amount of anti-CD28 mAb. As shown in Fig. 3B the percentage of CD4^+^ T cells producing IL-17 was significatively augmented with high doses of immobilized anti-CD3 mAb. Moreover, the strongest CD28 inhibitory signal for Th17 differentiation was delivered to T cells stimulated with high anti-CD3 concentration. This inhibition of Th17 differentiation could not be attributed to increased cell death. Indeed, the recovery of viable cells as well as apoptotic cell death rate were similar in CD3 and CD3 plus CD28 co-stimulated CD4^+^ T cells cultures from the early (48 h) to a late time point of restimulation (between 96 and 120 h) (Fig 3D).

### CD28 co-stimulation inhibits Th17 differentiation via IFN-γ and IL-2-dependent mechanisms

**Figure 4:**
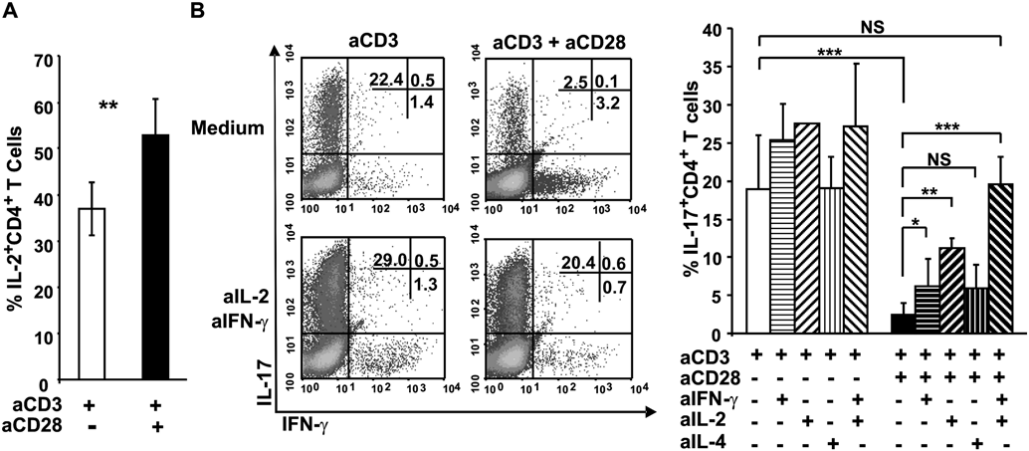
The CD28-driven inhibition of Th17 differentiation is mediated by IL-2 and IFN-γ production. Purified CD4^+^ T cells were stimulated with plate-bound anti-CD3 in the presence or the absence of soluble anti-CD28 mAb under Th17 polarizing conditions. (A) The percentages of IL-2^+^CD4^+^ T cells from 7 independent experiments (mean±SEM). (B) CD4^+^ T cells were cultured in the presence or absence of anti-mouse IL-4, IFN-γ or IL-2 alone or in combination. The data shown are representative of 1 experiment out of 3 (dot plots, left panel). The cumulative data from 2–6 independent experiments are shown in the right panel. P values were calculated using the two-tailed, paired Student's *t* test, *, P<0.05; **, P<0.01; ***, P<0.001.

CD28 signalling is reported to enhance cytokine production that includes IL-2, IL-4 and IFN-γ secretion in activated CD4^+^ T cells [20], we found that anti-CD28 mAb increased the proportion of IL-2-producing cells (Fig. 4A) as well as IFN-γ−producing cells in anti-CD3-stimulated CD4^+^ T cells (Fig. 1 and 3). Under Th17 polarizing conditions, neutralization of IL-2 or IFN-γ but not IL-4 partially overcame CD28 blockade and lead to increased IL-17 production. Nonetheless, the frequency of IL-17^+^CD4^+^ T cells remained inferior to that generated in the absence of CD28 co-stimulation. However, treatment with both anti-IL-2 and anti-IFN-γ mAbs restored IL-17 production and thus completely abrogated the effect of CD28 engagement (Fig. 4B).

**Figure 5:**
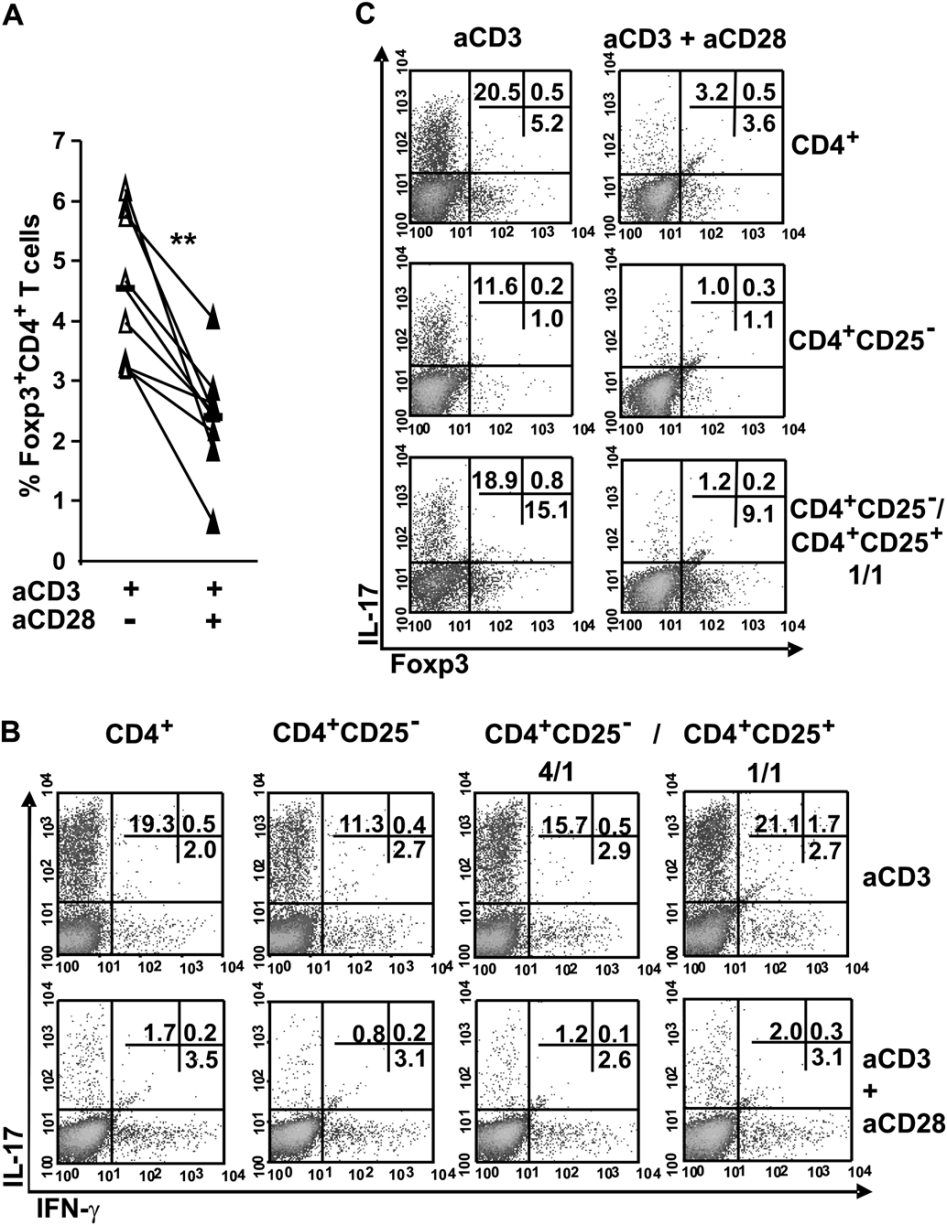
Inhibition of Th17 differentiation by anti-CD28 mAb is not correlated with a expansion of Foxp3^+^ T cells. Purified CD4^+^ T cells were stimulated with plate-bound anti-CD3 in the presence or the absence of soluble anti-CD28 mAb under Th17 polarizing conditions. (A) The proportion of Foxp3^+^CD4^+^ T cells. Data from 8 independent experiments are shown. (B and C) CD4^+^, CD4^+^CD25^−^ T cells and CD4^+^CD25^−^ T plus CD4^+^CD25^+^ T cells (at 1/1, 4/1 CD25^−^/CD25^+^ T cell ratios) were stimulated as described. Numbers indicate the percentage of IL-17 and IFN-γ-expressing CD4^+^ T cells (B) and IL-17 and Foxp3-expressing cells (C). One of 2 representative experiments is shown. P values were calculated using the two-tailed, paired Student's *t* test, **, P<0.01.

Since IL-2 partly mediated the inhibitory effect of anti-CD28 mAb on Th17 development, we postulated that the suppressive function of IL-2 operates through the expansion of Foxp3^+^ T cells. We here found that the percentage of Foxp3^+^CD4^+^ T cells was decreased rather than increased after CD28 signalling (Fig. 5A). We next directly addressed the contribution of Treg to the CD28-mediated suppression of Th17 differentiation by CD25^+^CD4^+^ T cell depletion and reconstitution experimental approaches (Fig. 5B). The IL-17 production was reduced in anti-CD3-stimulated CD4^+^CD25^−^ T cells. Co-cultures of CD4^+^CD25^−^ T cells and Treg at different CD4^+^CD25^−^/CD25^+^ T cells ratio enhanced Th17 differentiation. Of notes, Treg were recovered at lower proportion than expected at the end of the cultures that may be best explained by the overwhelming non Treg cell proliferation in the cultures (Fig. 2A and data not shown). Nonetheless, anti-CD28 mAb did not increase the frequency of IL-17^+^Foxp3^+^ or IL-17^+^Foxp3^−^ cells that were almost undetectable in CD25^+^ T cell-depleted polarised cultures (Fig. 5C **)**.

### Mature DC inefficiently support Th17 differentiation

**Figure 6:**
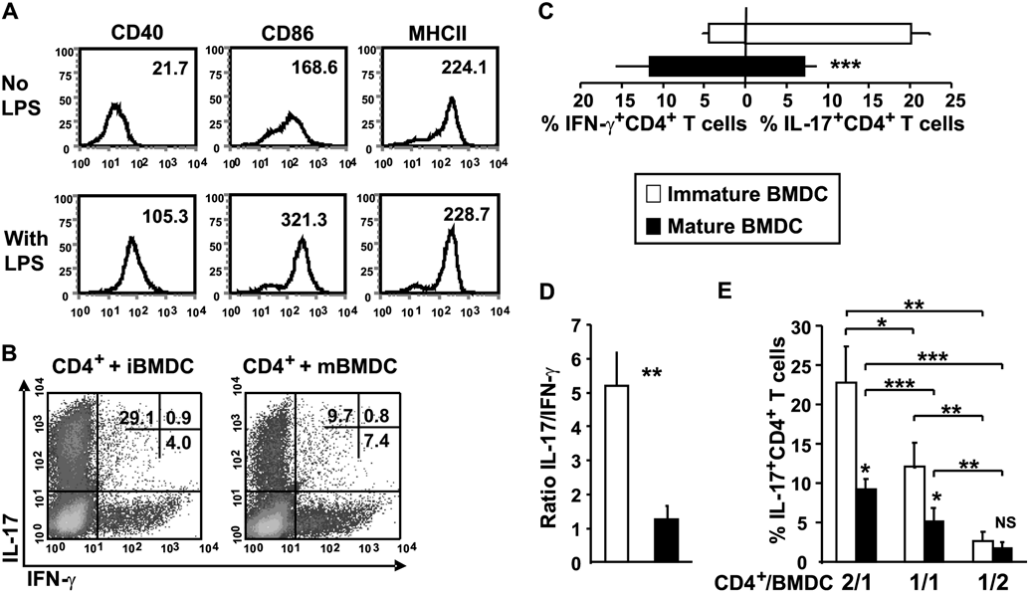
Mature BMDC inefficiently support Th17 differentiation. CD4^+^ T cells were cultured with immature or mature BMDC at a 2/1 CD4^+^/BMDC ratio in the presence of anti-CD3 under Th17 conditions. (A) BMDC were stimulated with LPS (1 µg/ml) and stained for CD40, CD86, or MHCII (IA/IE) expression. The mean fluorescence intensity are shown. Data are representative of one experiment out of three. (B) Cells were stained intracellularly for IL-17 and IFN-γ. Shown is one representative experiment out of 6. (C) Cumulative data of the percentage of CD4^+^IL-17 and IFN-γ positive T cells after restimulation as assessed by intracytoplasmic staining. Data represent the mean±SEM of 6 independent experiments. (D) The ratio of the percentage of IL-17^+^ to IFN-γ^+^ cells among CD4^+^ T cells. (E) CD4^+^ T cells (2.5×10^5^ cells) were cultured with immature or mature BMDC at three different CD4^+^/BMDC ratios (2/1; 1/1; 1/2) as described in A. Data represent the mean±SD of 4 independent experiments. P values were calculated using the two-tailed, paired Student's *t* test, *, P<0.05; **, P<0.01; ***, P<0.001.

The observations that Th17 differentiation can be suppressed by high levels of co-stimulation in an APC-free system led us to examine the ability of immature versus mature dendritic cells (DC) to drive Th17 development. Unstimulated (immature) or LPS-stimulated (mature) mitomycin C-treated bone marrow-derived DC (BMDC) (Fig. 6A) were cocultured with CD4^+^ T cells under Th17 differentiation conditions. As shown in Fig. 6B to E, the maturation status of the BMDC dictated the fate of Th polarization toward Th17. When CD4^+^ T cells were differentiated in the presence of mature BMDC, we observed a significant decrease in the percentage of Th17 cells and, as expected, an opposing effect on IFN-γ producing cells (Fig. 6B to D). Hence, immature BMDC induced higher frequencies of IL-17^+^CD4^+^ T cells when compared to their mature counterparts (Fig. 6E). Furthermore, increased CD4^+^ T cell proliferation was observed with mature BMDC under Th17 polarizing conditions containing TGF-β, suggesting that the inhibition of IL-17 secretion was unlikely related to a reduced expansion of CD4^+^ T cells (data not shown).

### CTLA4-Ig enhances Th-17 differentiation

**Figure 7:**
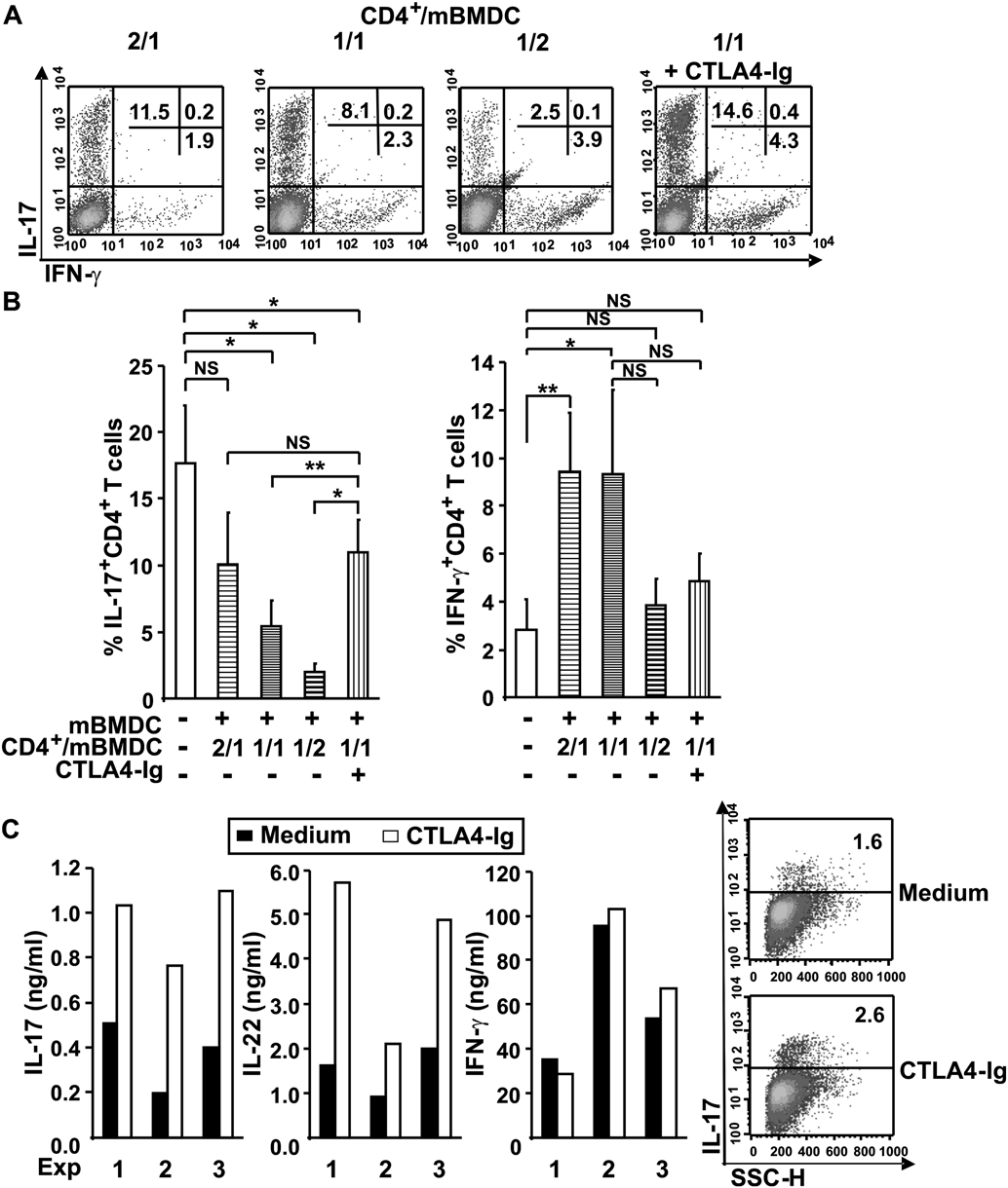
CTLA4-Ig favours Th17 development. (A) CD4^+^ T cells were cultured with mature BMDC at three different CD4^+^/mBMDC ratios (2/1; 1/1; 1/2) in the presence of anti-CD3 under Th17 conditions. CTLA4-Ig (20 µg/ml) was added to cultures with a 1/1 CD4^+^/mBMDC cell ratio. Cells were stained intracellularly for IL-17 and IFN-γ. Shown is one representative experiment out of 4. (B) CD4^+^ T cells were stimulated as in A. Data represent the mean±SD of 4 experiments. (C) Human naïve CD4^+^ T cells were co-cultured with activated monocyte-derived DC in the presence or absence of CTLA4-Ig (5 µg/ml), expanded in IL-2 and restimulated. IL-17, IL-22 and IFN-γ secretion was assessed in the culture supernatants and cells were stained for intracellular IL-17. Shown are 3 independent experiments (ELISA, left panel) and 1 representative out of 3 experiments (intracytoplasmic staining, right panel). P values were calculated using the two-tailed, paired Student's *t* test, *, P<0.05; **, P<0.01.

To evaluate the role of B7.1 and B7.2 co-stimulatory molecules on Th17 differentiation, we used CTLA4-Ig, which interrupts the CD28/B7 pathway. CD4^+^ T cells were co-cultured with graded numbers of mature-BMDC under Th17 polarizing conditions in the presence or absence of CTLA4-Ig. We found that CTLA4-Ig significantly augmented the frequency of IL-17^+^CD4^+^ T cells (Fig. 7, A and B). The proportion of IL-17-producing cells among CD4^+^ T cells co-cultured with mature BMDC at a 1/1 CD4^+^/DC ratio in the presence of CTLA4-Ig was similar to the percentage induced with half the number of DC and in the absence of CTLA4-Ig. Thus, lowering the mature DC/T cell ratio or interrupting B7 costimulatory pathways favoured Th17 differentiation.

Human monocytes and conventional DC, but not monocytes-derived DCs activated by microbial stimuli, induced Th-17 priming [21].We therefore determined whether CTLA4-Ig enhanced human Th17 differentiation in APC/T cells co-cultures. To this end, adult CD45RA^+^CD4^+^ T cells were cultured with mature monocyte-derived DC in the presence or absence of CTLA4-Ig but in the absence of exogenous cytokines or anti-IFN-γ mAb. Our data indicate that CTLA4-Ig significantly increased naïve CD4^+^ T cell differentiation into IL-17 and IL-22-producing cells, thereby confirming in human *in vitro* studies our observations in murine DC/CD4^+^ co-cultures (Fig. 7C).

## Discussion

In this study, we provide strong evidence for an inhibitory role of CD28 in the development of naïve CD4^+^ T cells into IL-17-producing T helper cells with no modulation of IL-17 expression in memory CD4^+^ T cells. We here demonstrate *in vitro* that ligation of CD28 with a monoclonal antibody results in inhibition of Th17 differentiation whereas CTLA4-Ig blockade enhances it during APC/T cell interactions. The extent of suppression of Th17 differentiation was regulated by the strength of TCR and co-stimulatory signals.

We explored some of the cellular mechanisms whereby CD28 engagement inhibited Th17 differentiation. CD28 co-stimulation decreased the frequency of Th17 proliferating cells without reducing their rate of cell division. Moreover, anti-CD28 mAb did not augment early or late apoptosis in anti-CD3-stimulated T cells cultures. Since CD28 signalling did not alter the recovery of viable cells at the end of the cultures, we propose that the decreased proportion of IL-17^+^ cells may result from an impairment of Th17 differentiation or an arrest of IL-17 production by Th17 proliferating cells. However, CD28 ligation during secondary cultures did not suppress but rather significantly enhanced IL-17 production in Th17 differentiated cells.

Although the importance of IL-6, TGF-β, IL-21 and IL-23 in Th17 development has been clearly established, less is known about physiological antagonists of Th17 responses. Th1 and Th2 cytokines negatively regulate the differentiation of Th17 cells. The addition of IL-12, IFN-γ or IL-4 suppresses either IL-23 or TGF-β and IL-6-induced differentiation of Th17 cells [5], [22], [23]. CD28 signalling enhances IFN-γ and IL-2 secretion in activated CD4^+^ T cells [20]. In agreement with these observations, we found that the enhancement in IFN-γ and IL-2-secreting CD4^+^ T cells was inversely correlated with the frequency of IL-17-producing cells. IFN-γ neutralization partially overcame CD28-mediated Th17 suppression. When combined, anti-IL-2 and anti-IFN-γ mAbs abrogated the effect of CD28 engagement. IL-2 and IL-27 inhibit Th17 differentiation [24], [25]. IL-27 can suppress the development of Th17 responses at least by inducing IL-10-producing cells [26]. The percentage of IL-10-secreting cells was low and not altered by CD28 co-stimulation (data not shown). IL-2 is a well-known T cell growth factor, yet, IL-2 deficiency is associated with severe multi-organ autoimmune disease characterised by the overproduction of IL-17 [24], [27]. These observations lead to the findings that IL-2 inhibits Th17 differentiation and promotes Treg differentiation [28].

In addition to the thymus-derived naturally occurring regulatory T cells (nTreg), naïve T cells can acquire Foxp3 expression and differentiate into induced Treg cells (iTreg) in peripheral tissues. TGF-β induces Foxp3 expression and iTreg cell differentiation from CD4^+^CD25^−^ T cells *in vitro* and *in vivo* [29], [30], [31]. Co-stimulation through CD28 impairs TGF-β mediated induction of Foxp3 expression in naïve T cells [32]. Our data indicate that CD28 co-stimulation decreased the percentage of Foxp3^+^ T cells under Th17 conditions. Although Th17 and Treg differentiation are controlled by reciprocal molecular pathways, naïve T cells activated with TGF-β and IL-6 can differentiate into Th17 cells in the presence of nTreg [6], [8], [33]. Our results are consistent with reports showing that Treg cells facilitate the differentiation of Th17 cells in a pro-inflammatory cytokine milieu [34]. Furthermore, regulatory T cells can be reprogrammed into Th17 cells [35]. T cells producing TGF-β that promote Th17 cell differentiation are absolutely required for the induction of EAE *in vivo* [36]. During an extracellular pathogen-driven inflammation, TGF-β (Treg) suppresses Th1 and Th2 differentiation and IL-6 impairs effector T cell responsiveness to Treg, allowing *de novo* differentiation of protective IL-17-producing T cells from naïve precursors. At the same time, CD28 co-stimulation may somehow limit Th17 differentiation to attenuate tissue damage. Nonetheless, since CD28 engagement alters the Th17/Th1 ratio toward Th1, under some circumstances it may aggravate autoimmunity [37], [38]. Taken together, in a Th17-promoting milieu, CD28 co-stimulation augments IFN-γ and IL-2 production and impairs Th17 polarization.

We next found that mature DC were less efficient than immature DC at driving Th17 polarization and that CTLA4-Ig favoured Th17 differentiation. These data strongly suggest that B7.1 and B7.2 are involved in the regulation of Th17 differentiation but do not exclude the role of additional pairs of co-stimulatory/inhibitory molecules. Hence, other co-stimulatory molecules may override the absence of B7 co-stimulation for T cell activation and differentiation into Th17 cells [39]. In support of our *in vitro* observations, several studies have demonstrated that interfering with B7 co-stimulatory pathways may alter the development or the course of Th17/Th1-associated autoimmune diseases *in vivo* [40]. Notably, B7.1/B7.2 deletion in SJL mice increases their susceptibility to EAE in contrast with the resistance to EAE observed in B7.1/B7.2^−/−^ C57BL/6 mice [41], suggesting that genetic background and B7 costimulatory molecules may dictate the outcome of the Th17 responses in absence of costimulation. In that regard, our unpublished observations indicated that anti-CD28 mAb significantly alter anti-CD3 stimulated CD4^+^ T cell differentiation into Th17 in BALB/c but not C57BL/6 mice (data not shown). In addition, while MOG induces EAE in wild type (CD28^+/+^) C57BL/6 mice, it induces immune-mediated meningitis (EAM) in CD28^−/−^ C57BL/6 mice, which is characterized by an infiltrate within the leptomeninges composed primarily of polymorphonuclear neutrophils [42]. T cell-derived IL-17 mediates the stimulation of neutrophil mobilization [43]. Also, B7-2-deficient NOD mice spontaneously develop autoimmune peripheral polyneuropathy [44]. Furthermore, inhibition of IL-17 prevents the development of arthritis in vaccinated mice challenged with *Borrelia burgdorferi*, while CD28 deficiency exacerbates joint inflammation upon *Borrelia burgdorferi* infection, resulting in the development of chronic Lyme arthritis [45], [46], [47]. Finally, reduced co-stimulation in either B7.1 or B7.2-deficient recipients resulted in a dramatic acceleration of colitis induction following transfer of CD4^+^CD45RB^high^ cells [48], [49]. Recently, the efficacy of CTLA4-Ig has been investigated in human clinical trials to prevent transplant rejection, and in the treatment of RA and psoriasis vulgaris [50]. We here showed that CTLA4-Ig significantly increased human naïve CD4^+^ T cell differentiation into IL-17 and IL-22-producing cells, thereby confirming in human *in vitro* studies our present observations in murine DC/CD4^+^ T cell co-cultures. These findings revealed the importance of B7.1 and B7.2 as negative regulators of human Th17 development. Considering human genetic variability, different individuals may have distinct requirements for costimulatory molecules to control Th17 development and the pathogenesis of autoimmune diseases. Thus, our data invoke caution when interfering with B7 co-stimulatory pathways for therapies, a concept largely debated in the literature [51], [52].

In conclusion, we identified an unexpected role for the B7/CD28 pathway in the regulation of Th17-associated inflammatory responses. These *in vitro* observations should be taken into consideration for the management of patients under B7-based immunotherapy for the treatment of autoimmune diseases and other immune-mediated disorders.
